# Research Progress on Extracellular Matrix-Based Composite Materials in Antibacterial Field

**DOI:** 10.34133/bmr.0128

**Published:** 2025-01-16

**Authors:** Dan Cai, Tuoqin Liu, Wei Weng, Xinhong Zhu

**Affiliations:** ^1^ Department of Orthopedics, The First People’s Hospital of Huzhou, First Affiliated Hospital of Huzhou University, Zhejiang 313000, China.; ^2^ Intensive Care Unit, People’s Hospital of Wuxing District, Wuxing District Maternal and Child Health Hospital, Huzhou, Zhejiang 313000, China.

## Abstract

Due to their exceptional cell compatibility, biodegradability, and capacity to trigger tissue regeneration, extracellular matrix (ECM) materials have drawn considerable attention in tissue healing and regenerative medicine. Interestingly, these materials undergo continuous degradation and release antimicrobial peptides (AMPs) while simultaneously promoting tissue regeneration, thereby exerting a potent antibacterial effect. On this basis, a variety of basic properties of ECM materials, such as porous adsorption, hydrophilic adsorption, group crosslinking, and electrostatic crosslinking, can be used to facilitate the integration of ECM materials and antibacterial agents through physical and chemical approaches in order to enhance the antibacterial efficacy. This article reviews the recent advancements in the study of ECM antibacterial materials, including the antibacterial function and antibacterial mechanism of free-standing ECM materials and ECM-based composite materials. In addition, the urgent challenges and future research prospects of ECM materials in the anti-infection industry are discussed.

## Introduction

It is common knowledge that many pathogenic microorganisms can proliferate at the surgical site when using biomaterials since they lack antibacterial properties [1,2]. The pathological changes brought on by graft infection can cause serious consequences and even death [3]. At present, the treatment of infections is importantly hampered by bacterial colonization and biofilm formation on implants [4,5], making it difficult for drugs and the body’s immune system to work effectively [6]. Furthermore, the spread of bacteria along implants can invade surrounding tissues and lead to more severe infections [7]. As a result, infection is often seen as a significant barrier limiting the further development and application of biomaterials.

The most effective antibacterial strategy for treating implant-related infections is antibiotic therapy [8], but in clinical settings, systemic administration is typically employed to achieve antibacterial effects, which directly contribute to the formation of drug-resistant bacteria [9]. Typical antibiotics such as penicillin and methicillin are no longer effective against resistant strains due to the development of antibiotic resistance [9,10]. Antibiotic resistance is thought to be a specific response of bacteria to the damage caused by antibiotics, which means that it cannot be avoided completely even if new antibiotics are created [11]. Due to the increased prevalence of antibiotic resistance, antimicrobial peptides (AMPs) are being increasingly explored as alternative antibacterial agents.

AMPs have the characteristics of broad antibacterial spectrum and immunomodulatory function [12]. Compared with traditional antibiotics, AMPs exhibit multiple modes of antibacterial activity, thus rarely causing antibiotic resistance [13]. The development of AMPs provides an effective direction to solve the problem of traditional antibiotic resistance, and is a new generation of antibacterial agent with great development prospects [14,15]. However, poor stability, short half-life, cytotoxicity, and high cost are key factors limiting the further clinical use of most AMPs [16]. The application of ECM materials provides an effective solution to the above problems.

The extracellular matrix (ECM) is composed of numerous structural and functional proteins, including collagen, fibronectin, proteoglycan, and glycosaminoglycan [17–19]. During tissue repair and remodeling, the components of the ECM will break down, and the various bioactive peptides released during this process are crucial for cell recruitment and healthy tissue recovery. They include bioactive peptides with antibacterial effects, which are beneficial for preventing ECM scaffold implant infections and offering protection when host inflammatory cell responses and humoral immune responses are activated [20,21]. Due to the gradual degradation of ECM materials in the body and their remodeling by host tissues, AMPs may continue to be released, providing sustained antibacterial effects [22].

**Fig. 1.**
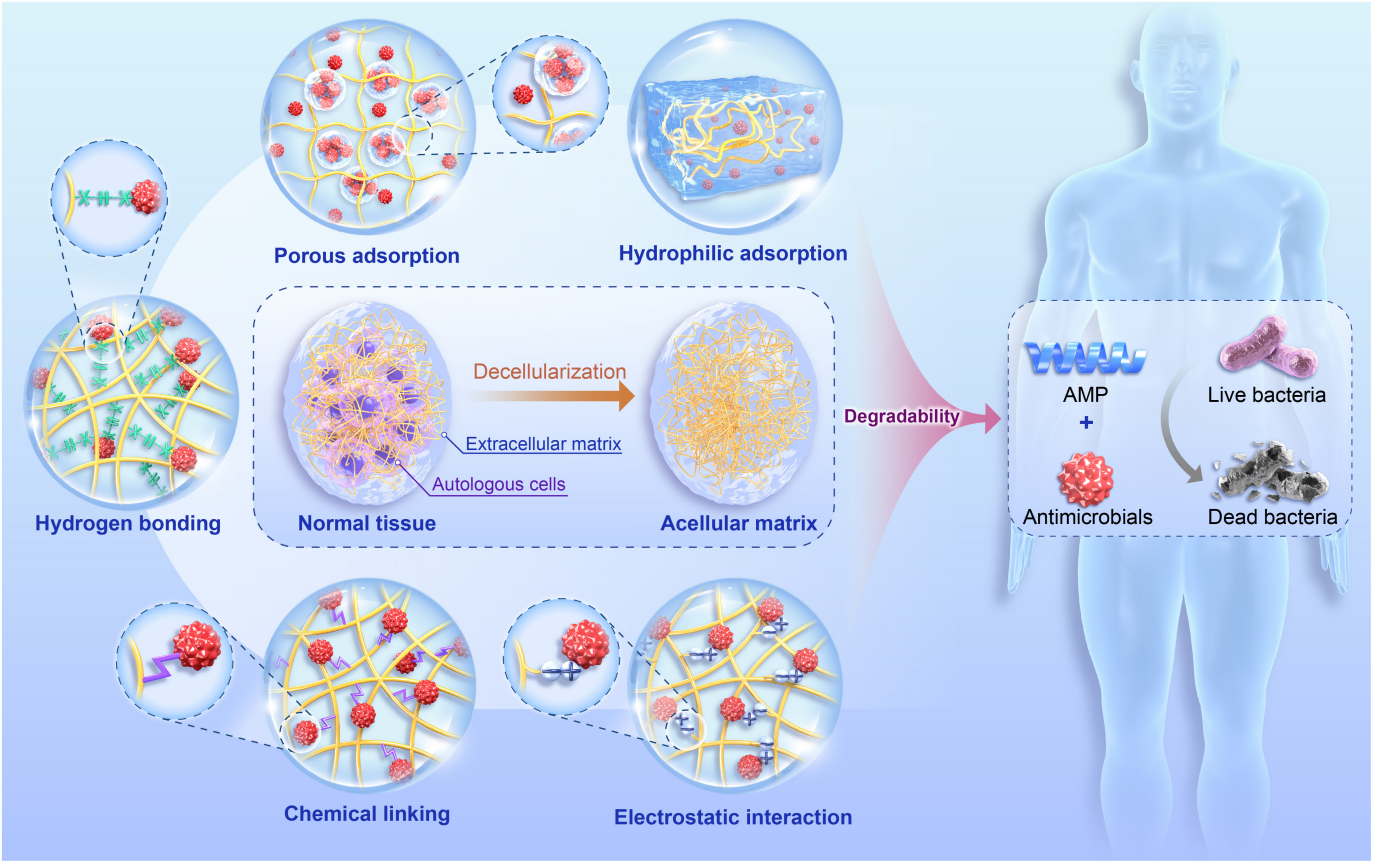
Schematic diagram of ECM materials combined with antibacterial agents to exert antibacterial function during biodegradation in vivo.

However, it is challenging to exert effective antibacterial effects on surrounding tissues in the early stages of ECM scaffolds that have not yet begun to degrade [22]. Therefore, ECM scaffolds have a limited capacity to resist infection. Related studies have shown that *Staphylococcus aureus* is more likely to adhere to ECM materials than synthetic ones [23–25]. This is why most researchers prefer to use undegraded ECM scaffolds as control groups for antibacterial studies [26]. ECM materials have the characteristics of porous structure, hydrophilicity, a large number of chemical groups, and surface negative charge [27–30]. These attributes are advantageous for the material to carry antibacterial medications and demonstrate antibacterial effects during the initial phase as shown in Fig. 1.

This review discusses the basic characteristics and antibacterial mechanism of ECM materials and explores strategies to enhance their antibacterial properties through the incorporation of exogenous antibacterial agents. The purpose of this review is to provide reference for the future research on ECM materials with antibacterial properties.

It should be noted here that the literature reference search for this review was conducted using the following databases: PubMed and Web of Science. Various combinations of keywords were employed, encompassing ECM scaffolds, degradation, AMPs, antibacterial agents, and antibacterial mechanisms. With a specific focus on antibacterial effects and mechanisms, an extensive analysis of the literature was performed to provide a more comprehensive depiction of composite antibacterial materials based on ECM.

## Basic characteristics of ECM materials

### Degradation properties

Degradability is an important property of ECM materials. Related researches have shown that host-mediated degradation of ECM materials is usually completed within 8 to 12 weeks [31]. Of course, the degradation rate can be effectively slowed down by employing physical or chemical crosslinking techniques. The continuous release of AMPs during degradation ensures sustained antibacterial effects [22]. Antibacterial drugs that are bound to the ECM materials through various methods are also released during degradation to enhance the antibacterial effect.

### Porous adsorption

Porosity is an important material characteristic in implants and tissue scaffolds [32]. The existence of pores within the material facilitates cellular infiltration, mechanical compliance, as well as the encapsulation and diffusion of drug formulations. Decellularization of tissues is a common strategy used in research to create scaffolds with a porous structure [33–35]. Drug delivery stands as a crucial application of the scaffold and is influenced by pore size. Highly porous biomaterials can effectively accommodate and release antibacterial drugs, thereby exerting antibacterial effects.

### Hydrophilic adsorption

ECM hydrogels are a 3-dimensional network structure of hydrophilic polymers with complex biochemical signal molecules similar to natural tissues and organs, which have the ability to retain water and easily encapsulate hydrophilic antibacterial drugs [28,36]. In the hydrogel structure, there are also a large number of pores and micro-pores, which can adsorb antibacterial drugs. When the hydrogel comes into contact with antibacterial drugs, the drugs will be adsorbed into the pores and micro-pores through physical adsorption or chemical bonding [37]. Over time, the drugs will dissolve and diffuse throughout the internal space of the hydrogel, eventually releasing into the external environment through a process of diffusion, osmosis, and biodegradation, thereby playing a role in antibacterial activity [38].

### Surface group crosslinking

The ECM scaffolds primarily consist of abundant ECM proteins, while the surface is characterized by a plethora of chemical groups that can be utilized to enhance the antibacterial performance of the scaffolds through various means of combining with antibacterial drugs [29]. These ECM scaffolds possess the capacity to establish hydrogen bonds with antibacterial agents, thereby eliciting antibacterial effects. Owing to the fragile nature of hydrogen bonds, they may be disrupted by alterations in pH, solvent composition, or temperature, leading to the release of the antibacterial agents [39]. Furthermore, chemical cross-linking can be employed to immobilize the antibacterial agents within the scaffold, and can be highly stable. Highly stable covalent linkages retain the drug until the scaffold degrades.

### Electrostatic interactions

ECMs contain a mixture of proteins, proteoglycans, and glycosaminoglycans. Among them, glycans have a high degree of negative charge [40]. Related studies have shown that the surface zeta potential of ECM stents is negatively charged, enabling them to attract and bind positively charged antibacterial drugs [41,42]. Fang et al. [41] cross-linked specific demineralized extracellular cancellous bone with vancomycin through electrostatic interaction and chemical bonding to play an after-effect antibacterial role.

## Antibacterial Properties Derived from ECM Materials

The majority of human body organs, such as the lungs, teeth, bladder, and gastrointestinal tract, communicate with the outside world directly; they are frequently exposed to a variety of bacteria but only rarely develop bacterial infections, which are closely related to the various ECM constituents [43,44].

Related studies have demonstrated antibacterial activity in the degradation products or extracts of ECM materials such as small intestinal submucosa [43], urinary bladder submucosa [44], and dental pulp and dentin [45] after acellular treatment. For example, Sarikaya et al. [43] obtained ECM extracts from the ECM of porcine small intestine submucosa and bladder submucosa through acetic acid digestion. Antibacterial tests showed that these ECM extracts had antibacterial activity against Gram-negative *Escherichia coli* and Gram-positive *S. aureus*. ECM extracts from dental pulp and dentin have been shown by Smith et al. [45] to have antibacterial activity against *Streptococcus mutans*, *Streptococcus oralis*, and fecal *E. coli*. Interestingly, Wandling et al. [46] processed ECM derived from bone marrow mesenchymal stem cells (BMMSCs) into decellularized nanoparticles that contained collagen, laminin, fibronectin, proteoglycan, glycoprotein, growth factor, and fibrillar protein. The BMMSC ECM nanoparticles could effectively inhibit the growth of *E. coli* and *Pseudomonas aeruginosa.* Therefore, ECM materials possess antibacterial properties, mainly due to the release of AMPs during the degradation of ECM content.

### Antibacterial effects of collagen VI and its derived AMPs

Collagen VI is the only ECM molecule that has been found to have direct killing properties to date [47,48]. It is a widely distributed ECM protein [49] made up of 3 distinct peptide chains: peptides 1 (VI), 2 (VI), and 3 (VI). Related studies have shown that collagen VI has antibacterial activity against Gram-positive respiratory pathogens and dose-dependent killing of streptococci, which can occur through membrane damage under physiological conditions [50]. Similarly, Abdillahi et al. [51] found that *Moraxella catarrhalis* (a Gram-negative respiratory pathogen) is a common cause of exacerbation of chronic obstructive pulmonary disease; it can adhere to collagen VI and subsequently be killed due to membrane instability at physiological pH levels and ionic strength. The protective abilities of the airway mucosa against potentially invasive oral pathogens and disease spread through the respiratory system are essential. An environment that enhances the innate antibacterial protection of the respiratory tract is provided by subcutaneous collagen VI, which has antibacterial properties [50,51].

To further investigate the antibacterial ability of collagen VI, Abdillahi et al. incubated Gram-positive bacteria *Streptococcus pyogenes* and *S. aureus* as well as Gram-negative bacteria *E. coli* and *P. aeruginosa* with purified collagen VI at 37 °C for 2 h for viable count determination. Research has shown that collagen VI possesses broad-spectrum antibacterial activity against the Gram-positive and Gram-negative bacteria mentioned above [49,51].

Interestingly, the AMP derived from collagen VI has killing activity against both Gram-positive and Gram-negative bacteria in vitro and in vivo; to some extent, this indicates that collagen VI can release AMPs during the degradation process, thereby exerting antibacterial effects as shown in Table 1 [49,50].

**Table 1.** Antibacterial active peptides derived from ECM protein

Protein		Peptide	Sequence	Ref.
Collagen VI		GVR28	GVRPDGFAHIRDFVSRIVRRLNIGPSKV	[49]
		FYL25	FYLKTYRSQAPVLDAIRRLRLRGGS	[49]
		FFL25	FFLKDFSTKRQIIDAINKVVYKGGR	[49]
		VTT30	VTTEIRFADSKPKSVLLDKIKNLQVALTSK	[49]
		SFV33	SFVARNTFKRVRNGFLMRKVAVFFSNTPTRASP	[49]
Laminin	α1	SRN16	SRNLSEIKLLSQARK	[55,58]
	α1	SRN29	SRNLSEIKLLSQARKQAASIKVAVSADR	[55,58]
	α1	KDF15	KDFLSIELFRGRVKV	[55,59]
	α1	SAV15	SAVRKKLSVELSIRT	[55,60,61]
	α3	SFM29	SFMALYLSKGRLVFALGTDGKKLRIKSKE	[56]
	α4	END11	ENDFMTLFLAH	[56]
	α4	END31	ENDFMTLFLAHGRLVYMFNVGHKKLKIRSQE	[56]
	α4	TLF20	TLFLAHGRLVYMFNVGHKKL	[57]
	α5	PPP25	PPPPLTSASKAIQVFLLGGSRKRVL	[55,63]
	α5	LGT25	LGTRLRAQSRQRSRPGRWHKVSVRW	[55,64]
	α5	RLR22	RLRAQSRQRSRPGRWHKVSVRW	[55,64]
	α5	PGR11	PGRWHKVSVRW	[55,57,64]
	α5	PGR12	PGRWHKVSVRWE	[56]
	α5	GLG27	GLGTRLRAQSRQRSRPGRWHKVSVRWE	[56]
	α5	RLV12	RLVSYGVLFFLK	[57]
	α5	GRW11	GRWHKVSVRWE	[57]
	β1	RIQ17	RIQNLLKITNLRIKFVKL	[60,62]
Fibronectin		QPP18	QPPRARITGYIIKYEKPG	[55,65]
Vitronection		AKK15	AKKQRFRHRNRKGYR	[55,62]

### Antibacterial effects of non-collagen-derived AMP

The ECM material is a complex composed of laminin, fibronectin, vitronection, and collagen, which plays an important role in the material’s function and biological activity [52–54]. Peptides formed from degraded laminin, fibronectin, and vitronection have been found to have antibacterial activity against Gram-positive *S. aureus*, Gram-negative *E. coli*, and *P. aeruginosa* [55–65].

Generally speaking, the strong correlation between heparin binding and the bactericidal properties of AMPs is widely accepted as common knowledge [49]. As these biodegradable active peptides have structural motifs associated with heparin affinity, they are endowed with antimicrobial properties [55,62]. Laminin contains a significant amount of heparin binding peptides, as shown in Table 1. Senyürek et al. [56,57] reported that the peptides derived from the LG4-5 module of human laminin α3, α4, and α5 chains exhibit antibacterial activity against Gram-positive and Gram-negative bacteria. In addition, they showed that these peptides can penetrate bacterial membranes and bind to bacterial DNA.

### Antibacterial mechanism and unique advantages of ECM materials

Throughout history, the ability of organisms to defend against microbial invasion has been a key to survival. AMPs are a part of innate immunity prevalent in all organisms and provide the first line of defense against microbial invasion [66]. AMPs have constantly been fighting against microorganisms, trying to kill them, while microorganisms are constantly evolving and putting up a tough fight against AMPs [67]. ECM materials derived from various organisms release many natural AMPs that enhance the body’s innate defense ability during the degradation process.

**Fig. 2.**
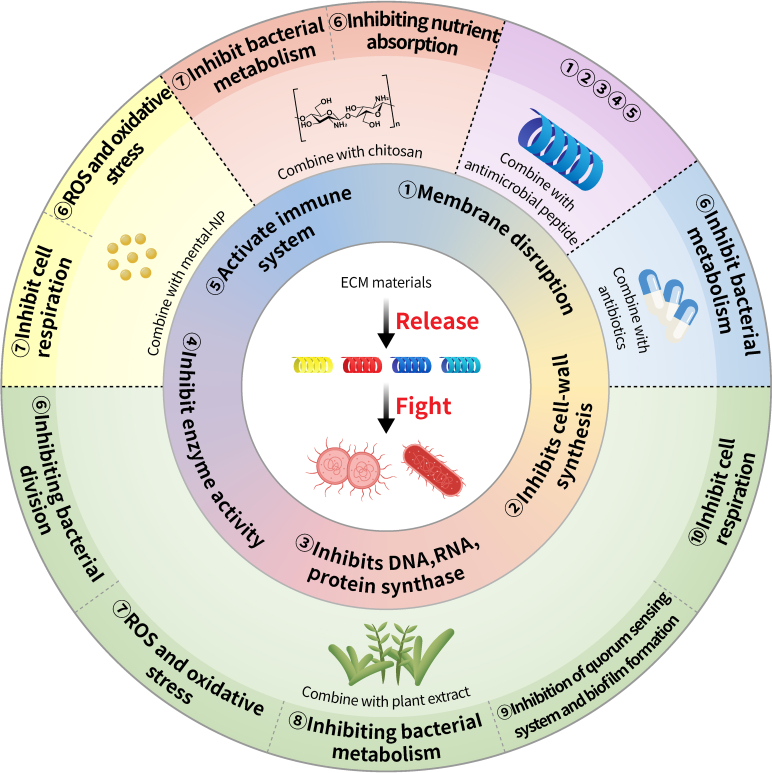
The antibacterial mechanism of AMPs combined with different antibacterial agents.

Natural AMPs exert their bactericidal effect by destroying cellular membrane integrity and binding with particular substances in the cytoplasm [68]. This interaction disrupts regular cellular metabolism, thereby conferring bactericidal properties. The confirmed nonmembrane destructive mechanisms of AMPs include inhibition of nucleic acid (DNA and RNA) synthesis, inhibition of protein synthesis, inhibition of enzyme activity, and destruction of cell walls [69]. In addition, AMPs can activate the host immune system to exert an indirect antibacterial effect by selectively inducing chemokine production in immune cells and recruiting other immune cells to the site of infection. Simultaneously, AMPs enhance antigen uptake and presentation while inhibiting apoptosis of neutrophils and macrophages [70]. AMPs that work through multiple pathways increase their antibacterial efficacy and reduce the tendency toward drug resistance as shown in Fig. 2.

It is widely acknowledged that AMPs, whether through genetic engineering or chemical synthesis, are currently difficult to produce at the industrial scale and do not meet the needs of the current market, thus limiting their application [71,72]. Regarding safety, some AMPs have hemolytic properties and low cytotoxicity in mammalian cells [73,74]. In terms of bioavailability, AMPs are essentially protein-degradable organisms [75]. However, the ECM material can effectively avoid the above shortcomings. In terms of production cost, the body can actively degrade ECM to release AMPs without laborious and costly industrial preparation technologies, including genetic engineering or chemical synthesis. In terms of safety, ECM itself has been extensively studied as a regenerative material with wide clinical applications [76]. In terms of bioutilization, AMPs are already the products of ECM degradation and require significant effort to undergo double degradation, which would compromise their structure [75,76]. ECM materials are continuously degraded, and AMPs are released as the body regenerates. The process is gentle and continues until the end of repair.

## Composite Antibacterial Properties of ECM Materials

The antibacterial effect of the ECM material is mainly related to the AMPs produced by its degradation. Therefore, it is difficult to effectively exert antibacterial effects in the early stages without material degradation. Holtom et al. [26] found the acellular submucosa of porcine small intestine lacks intrinsic antimicrobial properties. The presence of bacterial growth within the submucosa suggests that it may provide a conducive environment for bacterial proliferation. Therefore, it is essential to increase the antibacterial activity of ECM materials by introducing foreign antibacterial agents. ECM materials possess a porous structure, hydrophilic properties, a large number of chemical groups, and a surface negative charge characteristic. These attributes are advantageous for the material to encapsulate antibacterial drugs and exhibit antibacterial effects as shown in Fig. 1.

Combined drug therapy can provide a variety of antibacterial mechanisms as shown in Fig. 2, which can effectively avoid the occurrence of bacterial resistance. ECM composite materials not only produce antibacterial effects but also have other properties, such as oxidation resistance [77], increased mechanical strength [78], and hemostatic performance [79]. Here, we have organized a study on the composite antibacterial capabilities of metal ions, antibiotics, and other antibacterial compounds with ECM materials.

### ECM/metal composite synergistic antibacterial therapy

Metal items with antibacterial characteristics have been used by humans for a very long time, including silver dinnerware, bronze pottery, and silver acupuncture needles [80]. With the development of nanotechnology, the antibacterial potential of metal nanoparticles has been widely studied. The antibacterial activity of nanoparticles is attributed to their small size and high surface-to-volume ratio; in other words, the large surface area of nanoparticles enhances their interaction with microorganisms, thereby exerting a broad spectrum and highly efficient antibacterial activity [81–83]. Metal materials can be enclosed within ECM materials to enhance their antibacterial properties.

Due to their antibacterial, biocompatible, and helpful features for wound healing, silver nanoparticles (AgNPs) have received significant attention in recent years in medical applications [84,85]. Compared to the high toxicity of silver ions, AgNPs have a larger surface area-to-volume ratio and higher efficacy against bacteria. Most importantly, they are less toxic for humans [86].

**Fig. 3.**
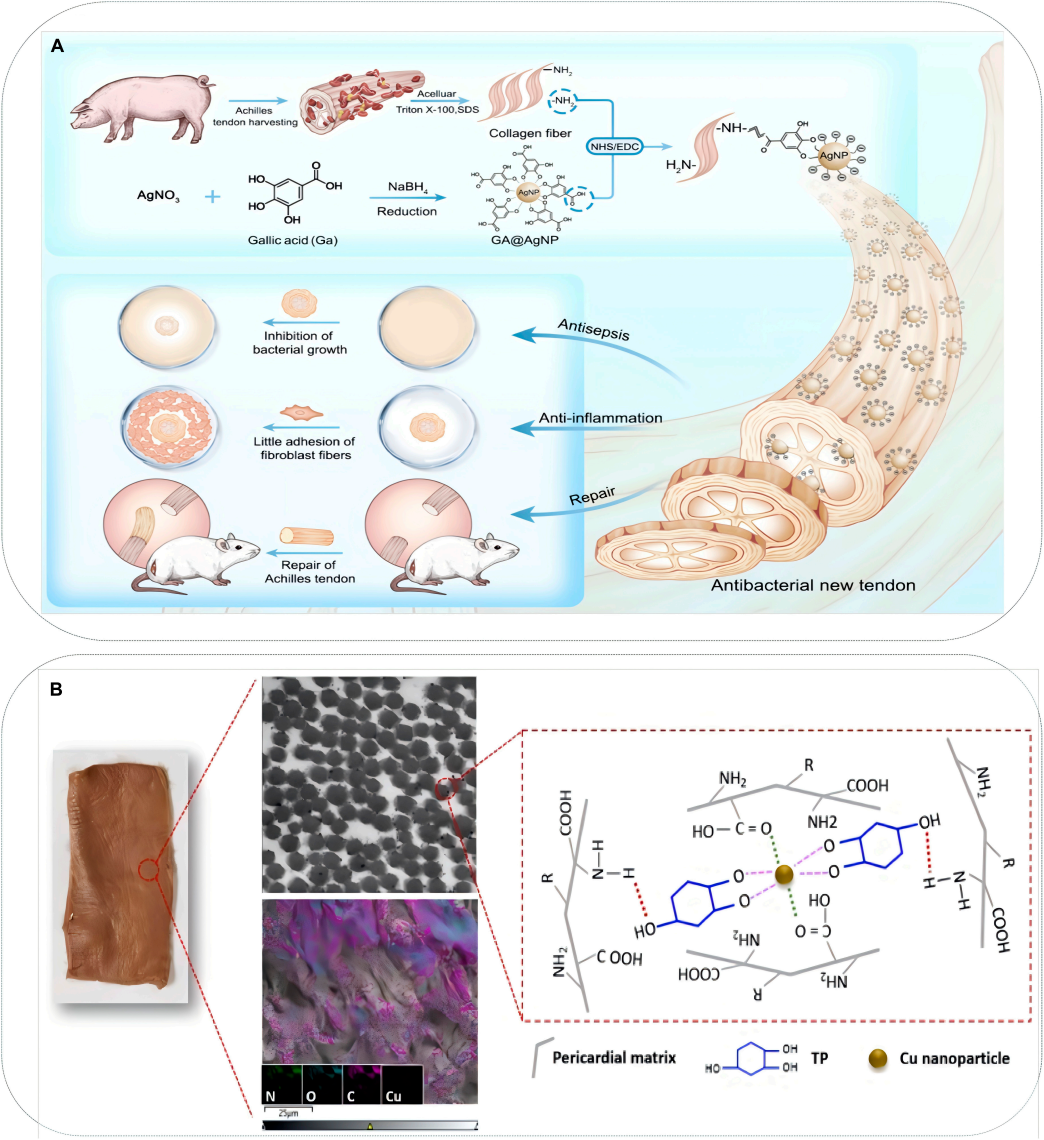
(A) Schematic illustration of the structure of GA-AgNPs and subsequent GA-AgNP-attached decellularized ECM for tendon reconstruction ([90] was adapted through open access permission). (B) Schematic representation of Cu@TP NPs and the resultant nanocomposite decellularized bovine pericardial scaffolds were named as Cu@TP-dBPs ([101] was adapted through open access permission).

AgNPs have multiple bactericidal mechanisms as shown in Fig. 2. Therefore, bacteria are less likely to develop resistance to AgNPs [87,88]. Tao et al. [89] combined silver nanoparticles with acellular dermal matrix (NS-ADM) for contaminated soft tissue defects; they found that NS-ADM retains a complete acellular structure and exhibits excellent biomechanical properties. When the concentration of nanosilver solution is less than 25 parts per million (ppm), NS-ADM has no cytotoxicity and shows strong antibacterial activity in vitro, making it safe for the treatment of abdominal wall defects. Chen et al. [90] synthesized AgNPs from gallic acid and silver nitrate, then attached with decellularized tendon via 1-ethyl-3-(3-dimethyl aminopropyl) carbodiimide (EDC)/N-hydroxysuccinimide (NHS) coupling, and formed micro- and nanoscale bilayer structures, thus endowing the corresponding tendon (DT-Ag) with good bactericidal properties and increased hydrophilicity as shown in Fig. 3A. Adhikari et al. [91] used acellular fish skin and biosynthetic AgNPs to successfully prepare a novel scaffold with antibacterial activity; AgNPs released by their scaffold could effectively kill *P. aeruginosa* and *S. aureus*. The incorporation of AgNPs into ECM scaffolds has been recognized as an effective strategy for achieving sustained antibacterial activity, as shown in Table 2. Consequently, the composite scaffold consisting of AgNPs and ECM has garnered increasing attention from researchers [92–94].

**Table 2.** Relates to antibacterial applications of ECM and antibacterial drug

Drug	ECM derived	Synthesis of the material	Combination mode	Target microorganism	Application	Ref.
AgNPs	Porcine skin	Acellular dermal matrix was immersed in the nano-silver solution	Physical mixture	*E. coli*, *S. aureus*, *P. aeruginosa*, and MRSA	Abdominal wall reconstruction	[89]
	Porcine tendon	Decellularized tendon was immersed into gallic acid-AgNP solution and crosslinked by EDC/NHS	Chemical crosslink	*E. coli* and *S. aureus*	Tendon reconstruction	[90]
	Fish skin	ECM scaffolds were soaked in the uniformly dispersed colloidal AgNP solution	Physical mixture	*P. aeruginosa* and *S. aureus*	Burn wound healing	[91]
Antibiotic	Porcine bone	Extracellular cancellous bone matrix and vancomycin were placed in EDC/NHS solution	Chemical crosslink Electrostatic interaction	*S. aureus* and *Enterococcus*	Infected bone healing	[41]
	Porcine small intestinal submucosa	Extracellular matrix envelope was hydrated in gentamicin solution	Physical mixture	*S. aureus*, *P. aeruginosa*, *S. epidermidis*, MRSA, *E. coli*, and *S. marcescens*	Prevention of cardiovascular implantable electronic device infection	[108]
		Decellularized ECM hydrated in vancomycin and gentamicin solution	Physical mixture	*S. aureus* and *S. epidermidis*		[109]
	Porcine skin	Acellular dermal matrix hydrogel was mixed with vancomycin	Physical mixture	*E. coli* and *S. aureus*	Infected wound healing	[110]
Chitosan	Porcine skin tissue	ECM, gelatin, and chitosan were mixed and crosslinked by EDC/NHS	Chemical crosslink	*E. coli* and *S. aureus*	Skin tissue engineering	[126]
	Porcine skin tissue	Thiolated chitosan, methacrylate ECM, and a plasma-treated polycaprolactone nanofiber dispersions were mixed	Photocrosslinking reaction	*E. coli* and *S. aureus*	Skin wound healing	[131]
	Porcine skin tissue	Prepared by adding acellular matrix and quaternized chitosan with gelatin as carrier	Physical mixture	*E. coli* and *S. aureus*	Repair of diabetic foot ulcers	[132]
	Porcine bone	Bone matrix was intermingled with oleoyl chitosan, and genipin was used for chemical crosslinking	Chemical crosslink	*E. coli* and *S. aureus*	Accelerate bone regeneration	[133]
	Porcine bladder submucosa	2-Hydroxypropyltrimethyl ammonium chloride chitosan, silk fibroin, and ECM graft were combined by blending and coaxial electrospinning	Physical mixture	*E. coli* and *S. aureus*	Reconstruction of the urethra	[134]
	Porcine dermal tissue	Oxidized 2-hydroxypropyltrimethyl ammonium chloride chitosan and ECM were mixed	Schiff base reaction	*E. coli* and *S. aureus*	Infected wound healing	[135]
	Human adipose	An upper layer of titanium dioxide-incorporated chitosan membrane and a sublayer of ECM sheet	Physical mixture	*E. coli* and *S. aureus*	Antibacterial wound dressing	[136]
AMP	Human placenta	Biological-based sponges made from decellulated human placenta loaded with CM11	Physical mixture	XDR *A. baumannii*	Antibacterial wound dressing	[149]
	Human dermis	Thrombin-derived host defense peptides GKY20 and GKY25 were added to acellular dermis	Physical mixture	*E. coli*, *P. aeruginosa*, and *S. aureus*	Surgery and wound treatment	[150]
Curcumin	Goat small intestine submucosa	Curcumin was embedded in decellularized goat small intestine submucosa	Chemical crosslink	*E. coli* and *S. aureus*	Skin tissue engineering	[163]
		ECM pre-gel was mixed with curcumin encapsulated eucalyptus oil-based nanoemulsion	Physical mixture	*E. coli* and *S. aureus*		[164]
Tea tree oil	Porcine skin	Two natural products quercetin and tea tree oil were added to modify pADM	Hydrogen bond	*E. coli* and *S. aureus*	Antibacterial wound dressing	[167]
Usnic acid	Porcine skin	Usnic acid was incorporated in ECM nanofibrous and poly(ε-caprolactone) scaffold	Chemical crosslink	*S. aureus*, *S. mutans*, *C. acnes*, *S. epidermidis*, and *C. albicans*	Infected wound healing	[173]
Honey	Ovine peritoneal	Ovine acellular peritoneal matrix, honey and ovine fetal skin extract were mixed	Physical mixture	*S. aureus* and *P. aeruginosa*	Infected burn wound healing	[175]
	Goat skin	Acellular goat-dermal matrix was dip coated in honey solutions	Hydrogen bond	*E. coli* and *S. aureus*	Infected wound healing	[181]

Copper is an excellent antibacterial agent with bactericidal and antibacterial activity [95–97]. Copper is cheaper and easier to obtain than silver. Therefore, the synthesis of copper nanoparticles is cost-effective [98]. In copper nanoparticles, antibacterial activity is associated with direct contact toxicity or the release of copper ions dissolved in the nanoparticles [99,100]. Li et al. [101] proposed using a green strategy to crosslink and functionalize decellularized bovine pericardial (dBP) scaffolds via the self-assembly of copper@tea polyphenol nanoparticles (Cu@TP NPs). The final scaffold was called Cu@TP-dBPs, and it had good biocompatibility, was beneficial to forming capillary-like networks, and effectively inhibited bacterial growth as shown in Fig. 3B. Copper is an essential trace element for the human body, and it helps to maintain homeostasis. Several adverse effects may occur if the intake of copper exceeds the tolerance range of the human body, including impaired lung, kidney, and liver function, as well as cellular toxicity due to oxidative damage [102,103].

### ECM/antibacterial drug synergistic antibacterial therapy

Antibiotics are widely used as antibacterial drugs, but their misuse has increased bacterial resistance, and the biological toxicity of some antibiotics cannot be ignored [104]. To avoid antibiotic misuse, researchers have linked antibiotics with implant materials to achieve long-term and controllable drug release. This strategy offers multiple benefits. First, local application can achieve efficient antibacterial effects in specific areas and is less likely to cause systemic toxicity. Second, a small amount of antibiotics are needed, resulting in better compliance and preventing potential antibiotic resistance [105–107].

High porosity levels are noted in natural ECM materials, allowing them to load antibiotics to exert antibacterial effects [108–110]. Sohail et al. [108] applied a biological ECM envelope, which consisted of multiple layers of ECM and originated from porcine small intestinal submucosa, in gentamicin solution to assess in vitro antibacterial action against 6 different bacteria. Related studies have demonstrated that the implantation of ECM capsules containing gentamicin results in a high local concentration of gentamicin, sustained local gentamicin concentration, and excellent in vivo bactericidal effects, thereby reducing the risk of systemic exposure to gentamicin. We prepared an injectable dermal ECM hydrogel derived from porcine dermal tissue for repairing skin defects. The hydrogel combined with vancomycin to induce hemostasis, accelerate antibacterial activity, and promote tissue repair. The antibacterial agent could promptly be released from the hydrogel within 1 h, which is shorter than the time required for bacterial infection and damage [112].

The physical binding of ECM materials with antibiotics leads to effective antibacterial effects, but it has rapid elution properties [111]. Furthermore, related studies have shown the exposure of bacteria to sub-inhibitory concentrations of antibiotics at the end of elution, leading to the generation of drug-resistant bacteria and the enhancement of bacterial virulence [112,113]. The “empty” ECM material will also lead to bacterial implantation when the antibiotics are depleted.

Most researchers have focused on loading antibiotics onto biomaterials through covalent modification, as this method will achieve sustained bactericidal effects by releasing antibacterial agents slowly and stably [114–116]. Fang et al. [41] created an antibacterial bone ECM scaffold by crosslinking the carboxyl group of cancellous bone acellular scaffold and the amino group of vancomycin using EDC/NHS. In acidic environments, this scaffold undergoes carboxylation, facilitating the rapid release of vancomycin, reaching up to 800 to 1100 μg/ml within a week, effective for early quick sterilization. After removing the infection from the acidic environment, the crosslinked vancomycin scaffold still demonstrated sustained drug release and contact bactericidal characteristics while suppressing osteoclast activity, promoting osteogenesis, and exerting immunomodulatory effects.

Topical administration of antibiotics can enhance bioavailability and minimize systemic adverse effects [117–121]. As a biodegradable drug delivery scaffold, ECM scaffold can optimize drug delivery efficiency through physical and chemical modifications, as well as facilitate sustained release for prolonged treatment.

### ECM/chitosan synergistic antibacterial therapy

Chitosan (CS) is a polysaccharide composed of D-glucosamine and N-acetylglucosamine [122]. CS possesses broad-spectrum antibacterial capabilities and considerable inhibitory effects on various microorganisms [123–125]. Xu et al. [126] mixed a porcine skin ECM solution with a solution containing a certain ratio of gelatin (Gel) and CS to prepare a ECM/Gel/CS solution. They then freeze-dried this solution in a 24-well plate to obtain a ECM/Gel/CS scaffold. The results indicate that adding CS improves the scaffold’s antibacterial properties to prevent wound infections.

Although CS contains free amino groups, it can only be dissolved in various dilute acids [126]. Furthermore, the strong hydrogen bonding between amino and hydroxyl groups makes the viscosity of CS solution relatively high and greatly limits its application [127]. CS contains -OH and -NH_2_ with certain chemical activity. Therefore, while preserving its beneficial properties, its water solubility can be improved by chemical modification or grafting copolymerization with other polymers [128].

**Fig. 4.**
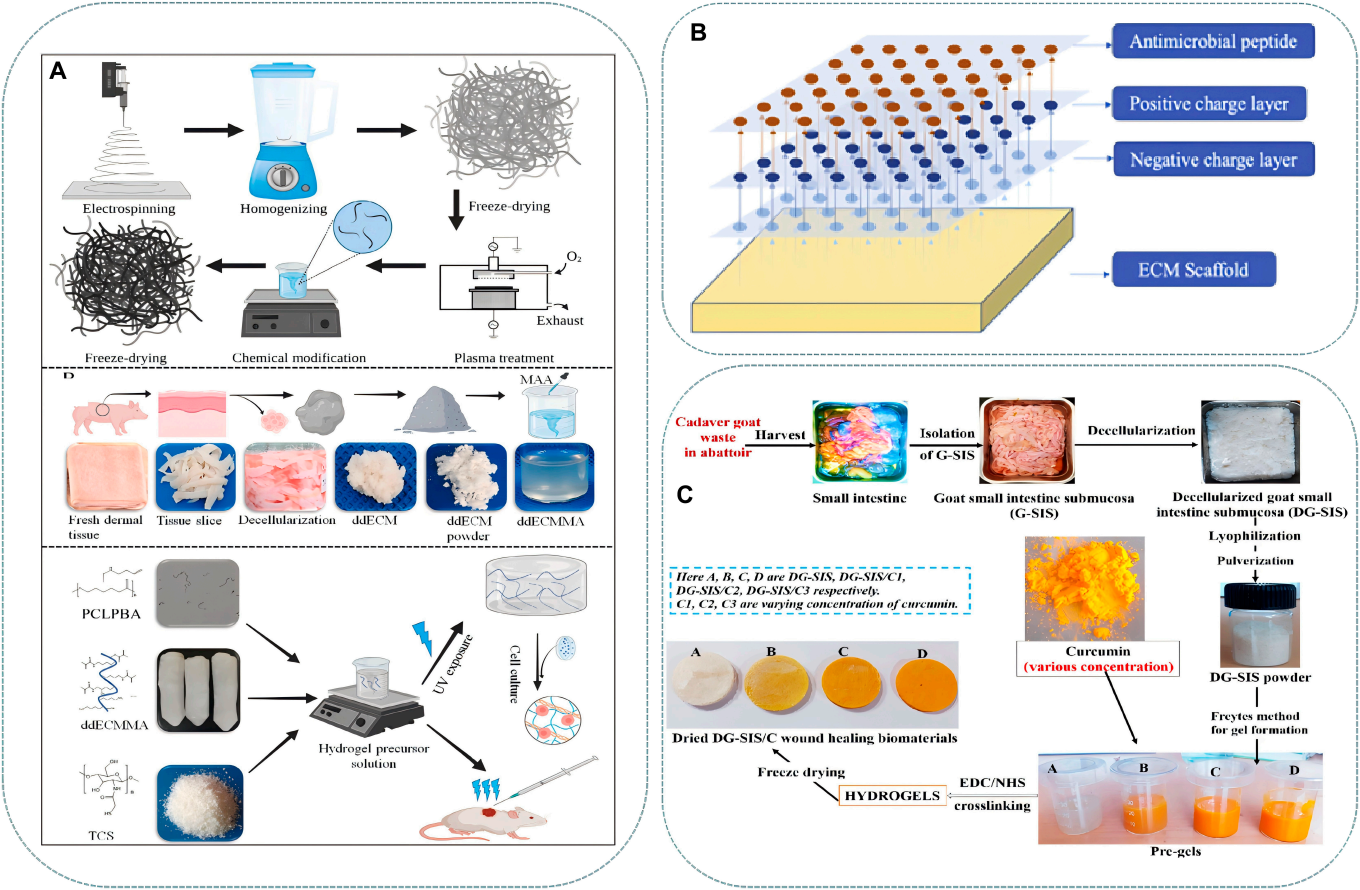
(A) Schematic image of PCLPBA preparation [3-buten-1-amine (BA)-modified polycaprolactone nanofiber with plasma treatment], decellularization, and hydrogel fabrication. Reprinted with permission from Elsevier Publisher Ltd. [131]. (B) Schematic of AMP formed ECM scaffold surface zeta potential. Reprinted with permission from Elsevier Publisher Ltd. [42]. (C) Systematic representation of fabricating the sustainable biomaterial scaffolds of decellularized goat small intestine submucosa and curcumin for wound healing and skin tissue engineering applications. Reprinted with permission from John Wiley and Sons [163].

For instance, quaternized CS (QCS) is an amino-modified CS derivative. Compared with CS, QCS has better water solubility and enhanced antibacterial activity due to the presence of many cations on its polymer chain [129]. Xu et al. [78] created a tissue-specific ECM-based bioink, which was assembled using an acellular ECM, Gel, QCS, and poly(ionic liquid)s. This scaffold could quickly kill both Gram-negative (*E. coli*) and Gram-positive (*S. aureus*) bacteria, had almost 100% antibacterial activity, and established a stable sterile environment within 7 days. Thiolated CS (TCS) showed excellent antibacterial effects against Gram-positive bacteria, Gram-negative bacteria, and fungi than CS [130]. Yu et al. [131] combined the decellularized dermal ECM with TCS to produce an ECM hydrogel that showed rapid gelation, good mechanical strength, and antibacterial and antioxidant properties as shown in Fig. 4A.

CS and its derivatives can be combined with ECM scaffolds in various ways to exert potent antibacterial effects, as demonstrated in Table 2 [131–136]. In addition to enhancing the antibacterial properties, the application of CS and its derivatives is beneficial to promote the clearance of reactive oxygen species, significantly reduce inflammatory reactions [77], strengthen hemostasis [79], prevent venous calcification [137], and improve the mechanical properties [138–140].

The potential of CS in the field of antibacterial [123–126] is significant; however, its immune side effects should not be underestimated. For instance, a study evaluating the biocompatibility of CS in mice demonstrated that CS scaffolds induced a typical acute inflammatory response characterized by mild neutrophil infiltration, which gradually subsided over time [141]. Relevant studies have shown that commercially available CS can elicit varying degrees of immune response when dissolved in solution [142]. Using purified, low endotoxin CS, it was determined that viscosity/molecular weight and degree of deacetylation within the ranges 20 to 600 cP and 80% to 97%, respectively, have no impact on CS’s immunoreactivity [143]. Consequently, prior to undertaking scientific research, it is recommended to select purified and low endotoxin CS in order to mitigate immune responses.

### ECM/AMP synergistic antibacterial therapy

AMPs are widely distributed in a variety of organisms. They belong to the innate immune response, can resist various pathogenic factors, and have a certain resistance to various microorganisms such as Gram-positive and Gram-negative bacteria, fungi, and viruses [144]. Numerous AMPs also have a killing effect on multidrug-resistant bacteria [145].

The introduction of foreign AMPs to enhance the antibacterial effect of ECM is an effective measure. Cecropin is the earliest discovered AMP in the world, and it has antibacterial activity against various Gram-negative and Gram-positive bacteria, fungi, and multiple drug-resistant strains [146–148]. Liang et al. [42] used CS, cecropin, and ECM scaffolds to form AMP-modified ECM scaffolds. The CS can form a positive charge layer on the surface of the ECM scaffold and change the surface characteristics of the material to bind with AMPs as shown in Fig. 4B. Research has shown that ECM scaffolds modified with AMPs possess good long-term antibacterial properties, and their performance is superior to ECM scaffolds soaked in antibiotics. Hamidabadi et al. [149] prepared a biological-based sponge, which was made from decellularized human placenta (DPS), and loaded it with different concentrations of AMPs. Antibacterial assays indicated that the DPS/AMPs had antibacterial behavior against extensively drug-resistant bacteria (XDR) *Acinetobacter baumannii* in a dose-dependent manner. The C-terminal peptide of human thrombin is produced in wound and fibrin in response to infection and inflammation. Kasetty et al. [150] coated human acellular dermis (hAD) with the thrombin peptides GKY20 (GKYGFYTHVFRLKKWIQKVI) and GKY25 (GKYGFYTHVFRLKKWIQKVI) and evaluated their in vitro activity against various bacterial strains characteristic for skin wounds. Research has shown that this scaffold has significant antibacterial effects against Gram-negative *E. coli*, *P. aeruginosa*, and Gram-positive *S. aureus*.

The stability of AMPs under physiological conditions is generally limited, making it challenging to deliver them to the site of infection and maintain their activity. However, it is possible to enhance their stability and antibacterial efficacy by loading AMPs onto ECM scaffolds as shown in Table 2.

### ECM/plant extract synergistic antibacterial therapy

Plant-based drugs have been cultivated to treat different diseases and prevent diseases caused by various microorganisms. Accordingly, an understanding of the therapeutic properties of different medicinal plants has spread in the scientific community. Using plant extracts as antibacterial agents could effectively address the widespread and imprudent use of synthetic antibiotics [151–153]. The World Health Organization recommends traditional drugs as the safest treatment for diseases caused by microorganisms [154,155]. Plant extracts are usually harmless, abundant in quantity, reasonably priced, biodegradable, multimechanism antibacterial as shown in Fig. 2 and sometimes exhibit broad-spectrum antibacterial activity [153,156].

Curcumin is an active natural polyphenol component of *Curcuma longa*, which has antibacterial, anti-inflammatory, antioxidant, antimutagenic, and liver-protective effects [157–159]. The use of curcumin is recognized as safe by the U.S. Food and Drug Administration [159]. Many scientists have investigated the abovementioned properties of curcumin for various uses, such as wound healing and antibacterial purposes. Curcumin also functions as an immunomodulator, facilitating the healing process of infected wounds by inhibiting virulence factors produced by pathogens and augmenting host-mediated immunity [160]. However, the therapeutic effects of curcumin are limited because this compound has low water solubility and is prone to rapid degradation [161,162]. Therefore, a carrier system is needed to encapsulate curcumin, protect it from degradation, and maintain its antibacterial properties. One such carrier could be an ECM material. Singh et al. [163,164] developed a novel biological scaffold for promoting wound healing by embedding curcumin into the decellularized small intestine submucosa of goat (DG-SIS). This scaffold can continuously release curcumin and inhibit bacterial growth at the wound site for a long time. In addition, curcumin enhances the antioxidant and biocompatibility properties of DG-SIS as shown in Fig. 4C.

Tea tree oil (TTO) is a volatile essential oil derived from the leaves of *Melaleuca alternifolia*, a plant valued for its antibacterial and anti-inflammatory activities [165–167]. Drugs and care products containing TTO are often used to treat infections or as preservatives and disinfectants [166]. Wang et al. [167] prepared a porcine acellular dermal matrix scaffold by adding 2 natural products as modifiers: quercetin (QCT) and TTO. The QCT was used to enhance the mechanical properties, and the TTO was used to enhance the antibacterial properties. Studies have shown that this scaffold has good antibacterial activity against both Gram-negative (*E. coli*) and Gram-positive (*S. aureus*) bacteria and has antibacterial activity levels of over 80%.

Usnic acid (UA), a dibenzofuran, was originally isolated from a lichen. It has been found to have antibiotic properties [168,169]. Many secondary lichen metabolites, including UA, protect lichen communities from other microorganisms. Related studies have demonstrated that UA can inhibit the growth of *Mycobacterium*, *Enterococcus*, and *S. aureus* [170,171]. The antibacterial mechanism of UA is mainly related to its inhibition of the synthesis of RNA and DNA [172]. However, the application of UA is limited by its unfavorable physicochemical properties, especially its poor water solubility [170]. Therefore, suitable carriers are required to load UA to benefit from its antibacterial effects. Chandika et al. [173] prepared a novel composite nanofiber poly(ε-caprolactone)/acellular ECM scaffold loaded with UA, in which UA was utilized as an antibacterial agent and wound healing promoter. This scaffold showed potent antibacterial activity against *Cutibacterium acnes*, *S. mutans*, *S. epidermidis* bacterial pathogens, and *Candida albicans* fungus pathogens. It also provided excellent inhibition rates for *Klebsiella pneumoniae* (60% to 68%) and *P. aeruginosa* (70% to 80%) biofilm formation.

Honey is a complex natural substance made by different bee species from the nectar of melliferous plants. Glucose oxidase from bee crops slowly degrades glucose into gluconic acid and hydrogen peroxide; the former compound reduces the pH of honey, while the latter helps to kill bacteria [174,175]. At wound sites, the lower pH of honey can effectively reduce protease activity, increase oxygen release from hemoglobin, and stimulate the activity of macrophages and fibroblasts. Additionally, the hydrogen peroxide disinfects the wound and encourages the production of vascular endothelial growth factors [176]. Furthermore, honey possesses immunomodulatory properties, exhibiting both pro-inflammatory and anti-inflammatory effects [177]. In the presence of low levels of inflammation, honey can stimulate the secretion of inflammatory cytokines and matrix metalloproteinase-9 (MMP-9). Conversely, when wound infection worsens and inflammation becomes difficult to control, honey can suppress the production of inflammatory cytokines and MMP-9 [177,178].

Honey not only exhibits broad-spectrum antibacterial activity against common wound infections but also demonstrates efficacy against antibiotic-resistant bacteria. Moreover, It can restore the efficacy of certain antibiotics against bacteria that had previously acquired antibiotic resistance [179,180]. Dhasmana et al. [181] accelerated the wound healing by combining acellular goat dermal matrix and honey (H-AGDM). The H-AGDM having 10% honey concentration is the best composition with good ultrastructure, antibacterial resistivity, biodegradability, biocompatibility, and anti-inflammatory response, which results in faster wound healing. The aforementioned plant extracts in an ECM scaffold can lead to better antibacterial performance and provide new possibilities for constructing more effective antibacterial materials as shown in Table 2.

In addition, the ECM material can simultaneously combine multiple antibacterial materials to achieve more efficient synergistic antibacterial effects. For example, Liang et al. [182] constructed a composite material of CS and tigecycline, which exhibited significant antibacterial activity against both Gram-negative and Gram-positive bacteria due to the synergistic effects of CS and tigecycline. Bankoti et al. [183] used CS as an ion crosslinking agent and iodine-modified 2,5-dihydro-2,5-dimethoxy-furan as a covalent crosslinking agent to double crosslink acellular dermal matrix. Both CS and iodine possess antibacterial effects, and bacteria are less likely to develop resistance to iodine. In addition, Majumder et al. [119] produced a novel minocycline/rifampin tyrosine-coated noncrosslinked porcine angular matrix to protect the device from microbial colonization for up to 7 days. Therefore, it is evident that composite scaffolds can effectively improve the antibacterial effects of ECM and have broad research applications.

## Conclusion and Future Perspectives

ECM materials have advantages, including good cell compatibility, biodegradability, and the ability to induce tissue regeneration. However, their antimicrobial properties have received little attention. In this review, we reported the current advances and development prospects of ECM-based antibacterial materials. The degradation of ECM materials resulted in the release of various AMPs rather than the release of a single AMP. The ECM material originates from various biological tissues, and the released AMPs can strengthen innate immunity to a certain extent. On this basis, ECM scaffolders can be chemically coupled or physically mixed with various other antibacterial substances, including AgNPs, CS, and antibiotics, to improve the antibacterial activity of the materials.

By summarizing the research progress of ECM materials combined with antibacterial drugs, it is evident that there are variations in the sources and types of ECM materials utilized, as well as differences in the types of antibacterial drugs employed and their combinations. Consequently, the application potential of composite materials is highly adaptable. First, diverse sources and types of ECM materials can be utilized for targeted anti-infection and repair purposes in specific infected tissues such as bones, cartilage, skin, muscles, and tendons. Second, ECM materials are available in various forms, such as hydrogels, powders, sponges, and 3D printed structures. This versatility allows for their application on different types of infected tissues, particularly those that are irregular or deep. Finally, depending on the level of wound contamination present, different antibacterial methods can be selected to achieve corresponding antibacterial effects. In aseptic wounds, the ECM can release a range of AMPs during degradation processes that serve preventive and antibacterial functions. In cases where suspected infections are involved, covalent bonding between their own chemical groups within the ECM and antibacterial drugs can result in long-lasting and more potent antibacterial effects being achieved. For infected wounds specifically, porous natured ECM materials can physically combine with antibacterial drugs, thereby causing the explosive release of antibacterial drugs and playing a powerful antibacterial role. On this foundation, covalent modifications have been implemented to attain sustained antibacterial efficacy. Therefore, using ECM materials with potential antibacterial properties is a promising treatment method.

Of course, there are still some challenges to overcome for antibacterial materials based on ECM. The main challenge so far is how to effectively control the degradation rate of ECM materials to achieve antibacterial effects. This is closely related to the complex structure of ECM materials themselves. Further research on the internal structure and polymerization mode, as well as physical or chemical modification of ECM materials, is essential to achieve better antibacterial effects. As an important structural and functional component of antibacterial composite materials, ECM has broad research prospects and enormous application value in the future. We believe that this review can provide new ideas and insights for biomedical researchers interested in creating new, highly effective antibacterial materials and clinical practitioners looking for alternative therapies to manage resistant bacteria.

## Data Availability

No data were used for the research described in the article.
